# Supplementary material for: Testicular somatic cell-like cells derived from embryonic stem cells induce differentiation of epiblasts into germ cells
Source: Commun Biol. 2021 Jun 28;4:802. doi: 10.1038/s42003-021-02322-8 (PMC8239049; doi:10.1038/s42003-021-02322-8)
Supplement: Supplementary file 2 — Supplementary Information [file 42003_2021_2322_MOESM2_ESM.pdf]

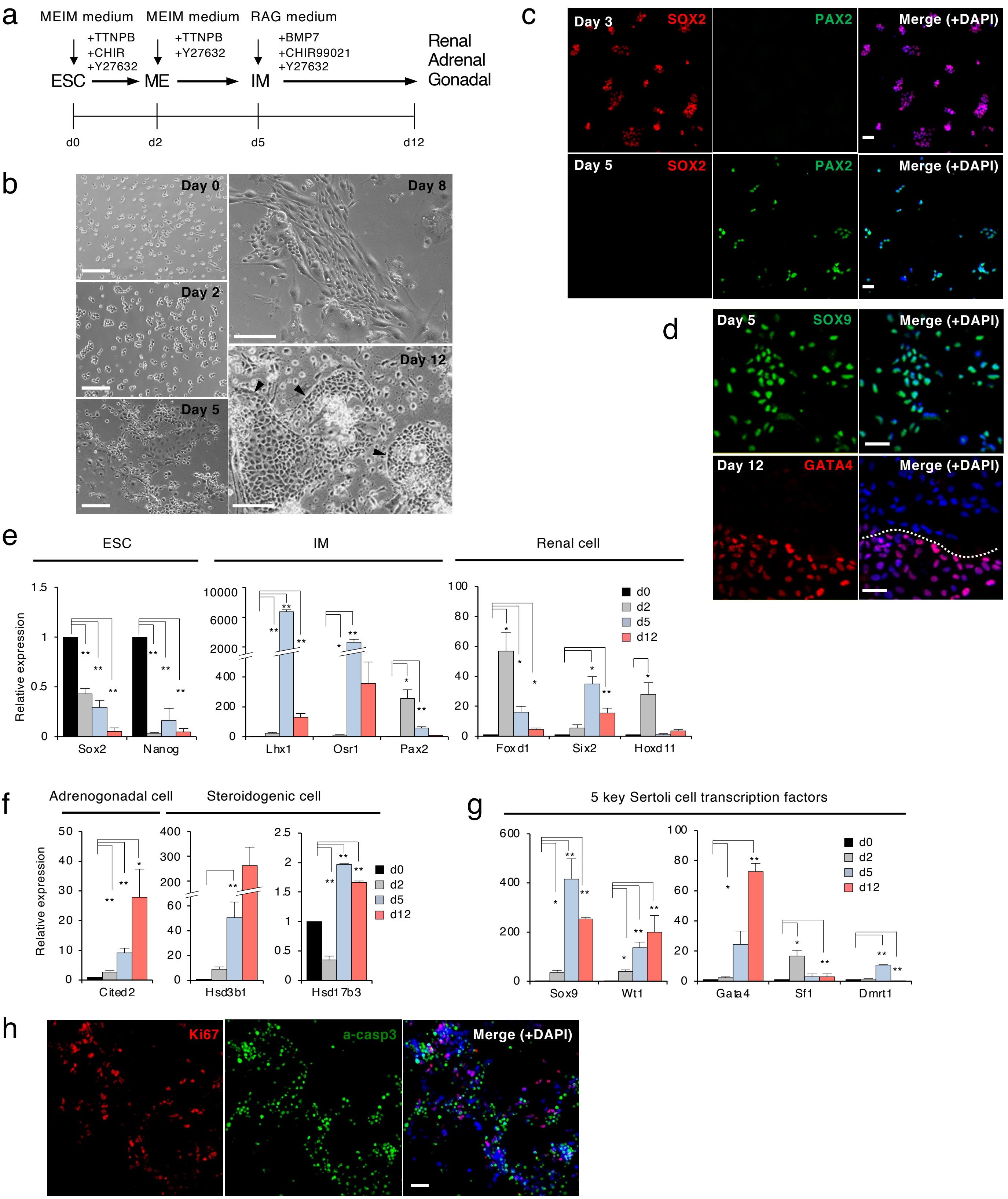

Supplementary Fig. 1. 3-step induction for differentiation of ESCs into SCLCs. a, Experimental design for sequential differentiation from ESCs to SCLCs via mesoendoderm (ME) and intermediate mesoderm (IM)–like cells. **b,** Morphological changes throughout differentiation process. Mesenchymal-shaped cells predominantly appeared between day 5 and 8, and the majority of them were turned into epithelial-shaped cells by day 12. Arrowheads indicate the basement membrane. **c,d,** Immunocytochemistry for a pluripotent marker, SOX2 (red), intermediate mesoderm marker, PAX2 (green), Sertoli cell markers, SOX9 (red) and GATA4 (red), on cells differentiated at day 0, day 5 and day 12. The dotted line demarcates the basement membrane of SCLCs. **e-g,** Quantitative RT-PCR analyses for the expression of ESC (*Sox2* and *Nanog*), IM (*Osr1*, *Lhx1* and *Pax2*) and renal (*Foxd1*, *Six2* and *Hoxd11*) markers (**e**), adrenogonadal (*Cited2*) and steroigogenic (*Hsd3b1* and *Hsd17b3*) markers (**f**), and 5 key transcription factors required for Sertoli cell differentiation (*Sox9*, *Gata4*, *WT1*, *Sf1* and *Dmrt1*) (**g**). Fold expression changes relative to day 0 were calculated by ΔΔCt method with standard error. 4 biological replicates, each of which had 3 technical replicates, were used for two-sided t-test. **P< 0.01, *P< 0.05. h, Immunocytochemistry for a cell proliferation marker, Ki67 (red), and apoptotic marker active-caspase 3 (green), on cells differentiated at day 5. DAPI (blue) for nuclear staining. Scale bar, 50 µm.

a

Condition		1st medium base	1st medium supplements	2nd medium base	2nd medium supplements
I	1	MEIM	CHIR + TTNPB	RAG	
	2	MEIM	CHIR + TTNPB	RAG	BMP7
	3	MEIM	CHIR + TTNPB	RAG	FGF9
	4	MEIM	CHIR + TTNPB	RAG	BMP7 + FGF9
II	1	MEIM	CHIR + TTNPB	RAG	TTNPB
	2	MEIM	CHIR + TTNPB	RAG	TTNPB + BMP7
	3	MEIM	CHIR + TTNPB	RAG	TTNPB + FGF9
	4	MEIM	CHIR + TTNPB	RAG	TTNPB + BMP7 + FGF9
III	1	MEIM	CHIR + TTNPB	RAG	CHIR
	2	MEIM	CHIR + TTNPB	RAG	CHIR + BMP7
	3	MEIM	CHIR + TTNPB	RAG	CHIR + FGF9
	4	MEIM	CHIR + TTNPB	RAG	CHIR + BMP7 + FGF9
IV	1	MEIM	CHIR + TTNPB	RAG	TTNPB + CHIR
	2	MEIM	CHIR + TTNPB	RAG	TTNPB + CHIR + BMP7
	3	MEIM	CHIR + TTNPB	RAG	TTNPB + CHIR + FGF9
	4	MEIM	CHIR + TTNPB	RAG	TTNPB + CHIR + BMP7 + FGF9

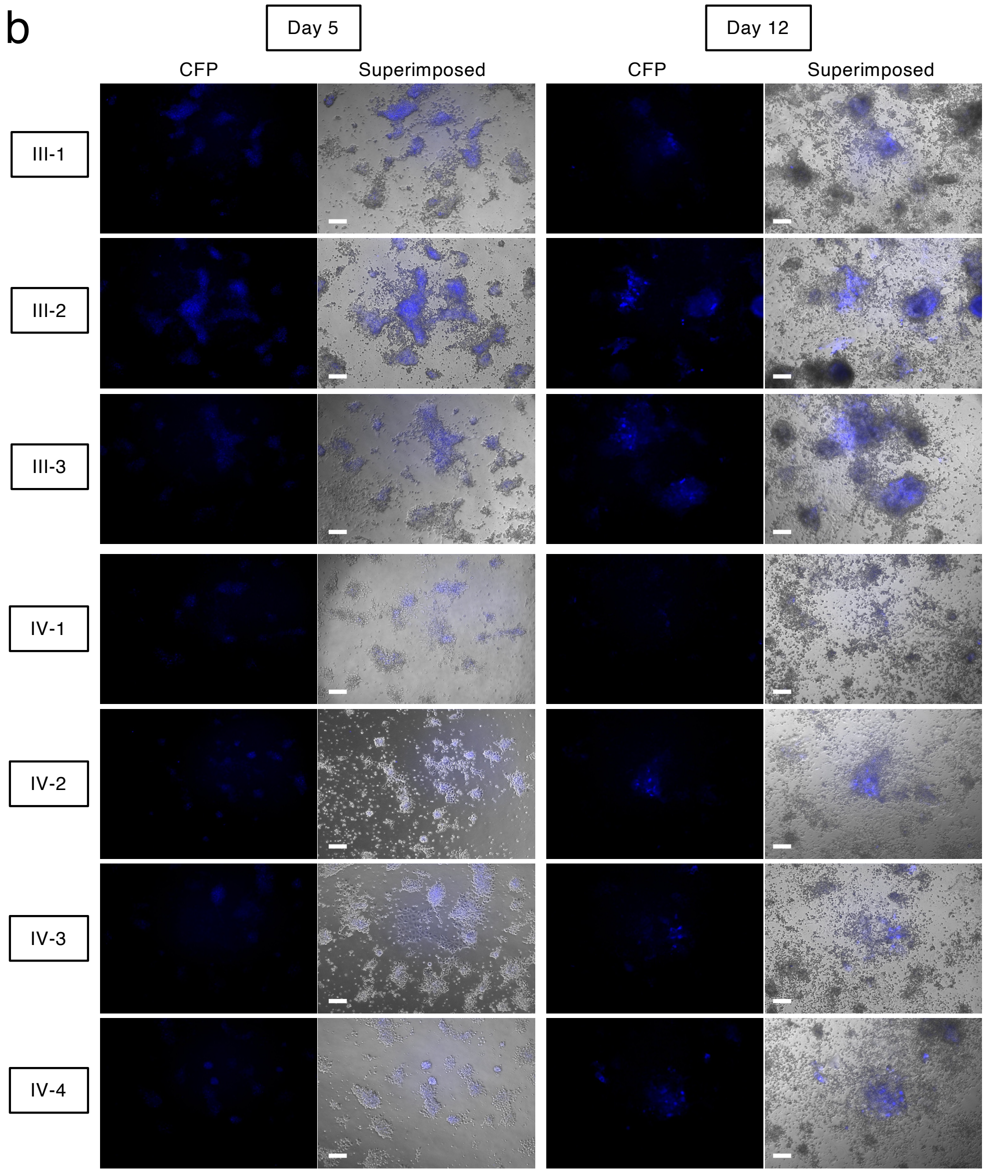

Supplementary Fig. 2. Optimization of SCLC differentiation. a, Summary table of culture conditions for SCLC differentiation with different combinations of growth factors (BMP7 and FGF9) and small molecules (CHIR99021 and TTNBP). **b,** Bright field images and those superimposed with CFP-fluorescent images are shown. Scale bar, 50 µm.

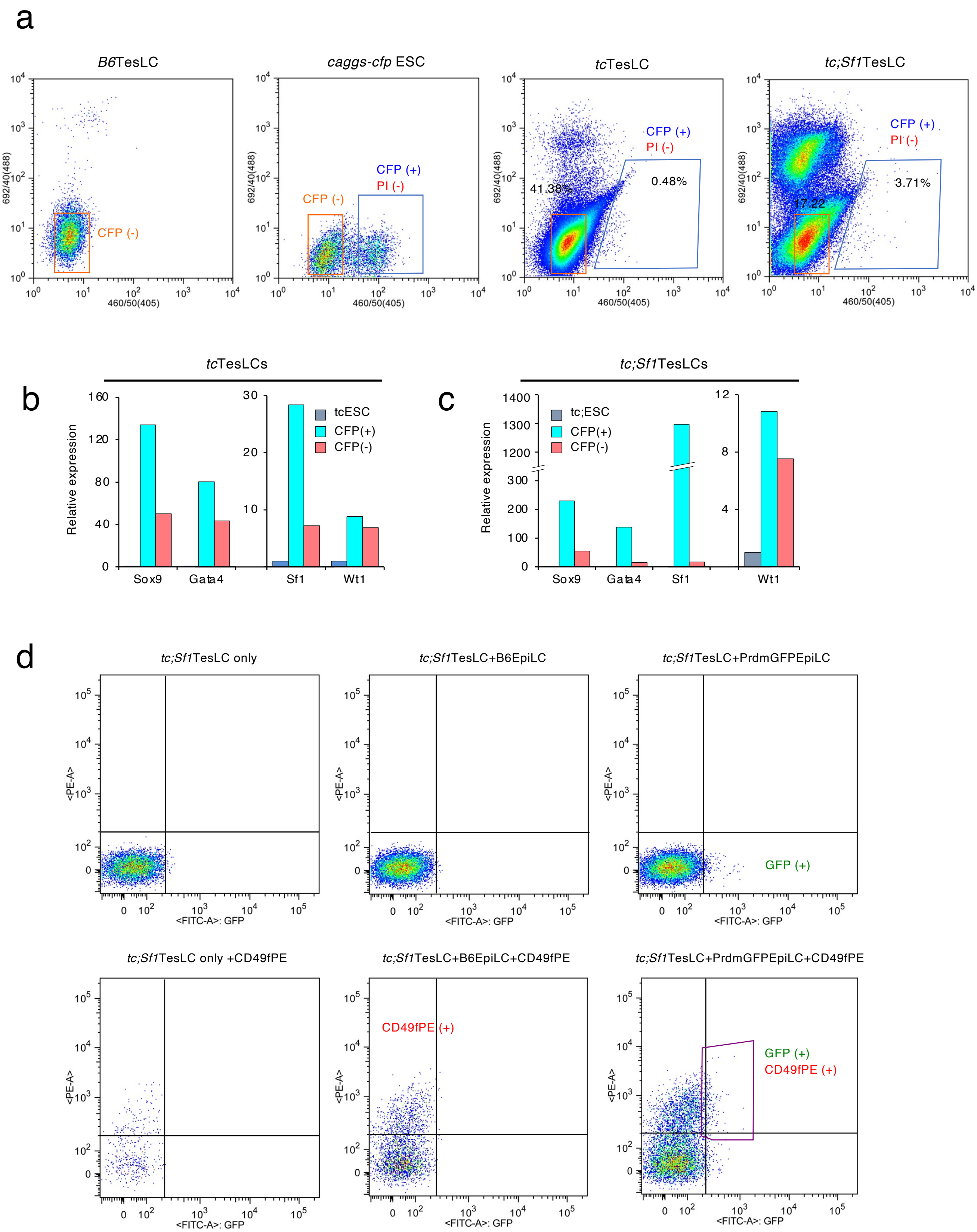

Supplementary Fig. 3. Flow cytometry analysis for SCLCs and tod-PGCLCs. a, Induction efficiency is indicated by percentages of viable CFP-positive cells in the singlet population from dissociated *tc*TesLCs and *tc;sf1*TesLCs. Non-fluorescent TesLCs derived from wild-type C57BL6 ESCs (*B6*TesLC) and *caggs-cfp* ESCs constitutively expressing CFP (*caggs-cfp*) were used as negative and positive controls, respectively. Propidium iodide (PI) was used for labeling dead cells. **b.c,** Relative expression of key Sertoli cell transcription factors in CFP-positive SCLCs and CFP-negative cells, compared to parental ESCs. n = 3 technical replicates. **d,** Isolation of tod-PGCLCs with GFP and CD49f-PE signals from testicular organoids composed of *tc;Sf1*TesLCs + *Prdm1-gfp*EpiLCs (bottom, right). After compensating signals between CFP derived from *tc;Sf1*TesLCs and GFP derived from *Prdm1-gfp*EpiLCs, gating for GFP-positive PGCLCs was optimized by comparison of *tc;Sf1*TesLCs only (top, left) and *tc;Sf1*TesLCs + B6EpiLCs (top, middle). To optimize gating for CD49f-positive cells, they were also reacted with CD49f-PE antibody or SSEA1-PE antibody as positive (bottom, middle) and negative (bottom, left) controls. DAPI was used to eliminate dead cells.

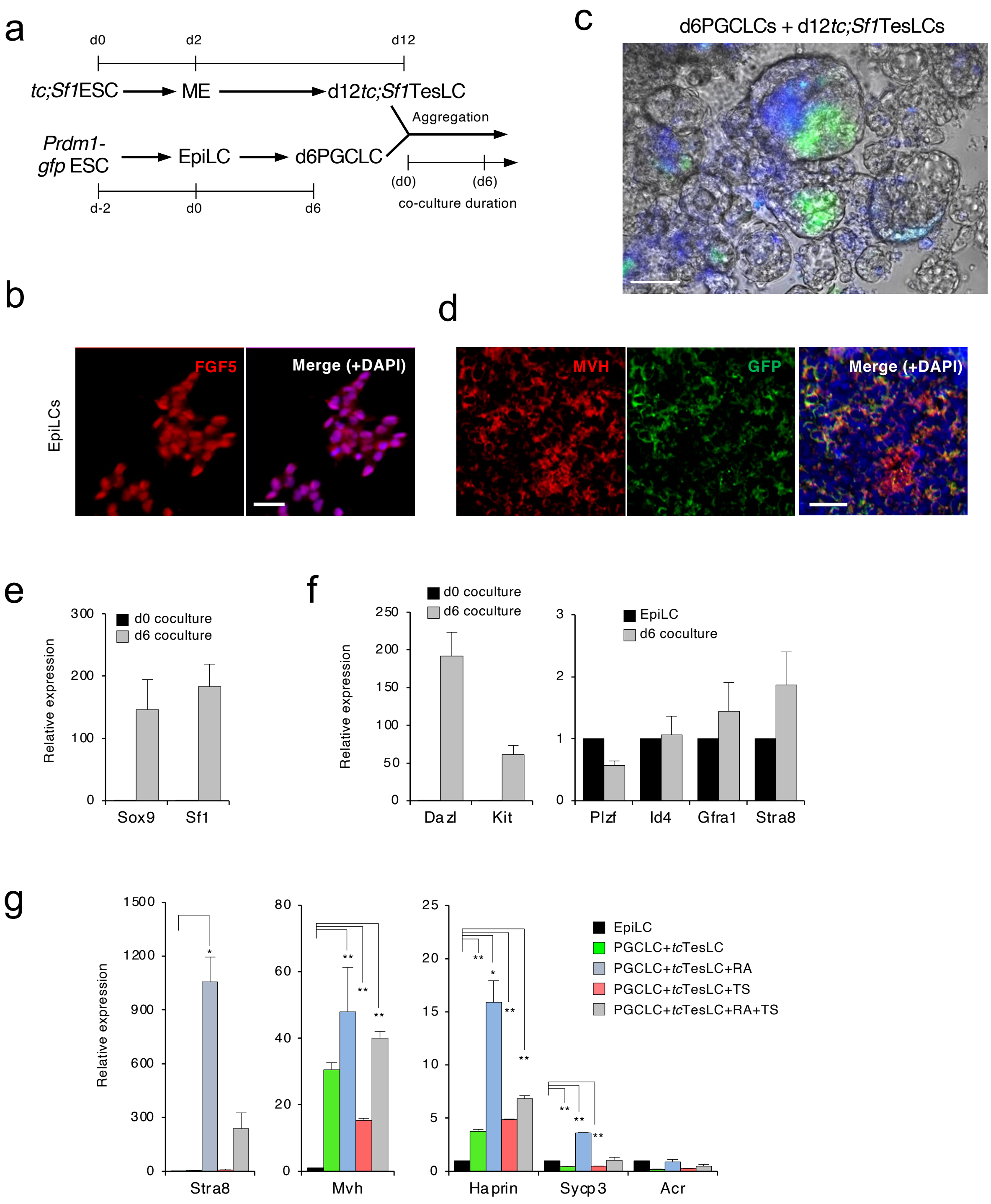

Supplementary Fig. 4. Aggregation co-culture of TesLCs and PGCLCs. a, Schematic outline of aggregation co-culture of d12TesLCs and d6PGCLCs. **b** Verification of the quality of EpiLCs by FGF5 expression. Almost 100% of cells were FGF5-positive. **c,** Superimposed GFP and CFP-fluoresce on bright-field images of day 6 co-culture derived from the aggregation of CBF-treated d12*tc;Sf1*ESCs and d6*Prdm1-gfp*PGCLCs. **d,** Maintenance of PGCLCs in the aggregation confirmed by co-expression of GFP (green) and MVH (red). **e.f,** Relative expression of a Sertoli cell marker *Sox9*, an early PGC marker *cKit*, a late PGC/gonocyte marker *Dazl*, spermatogonial stem cell markers *Gfra1*, *Plzf* and *Id4*, and meiotic marker *Stra8* in the aggregate. The expression levels of these genes in day 6 of aggregates relative to those in day 0 of aggregates or parental EpiLCs were indicated with standard errors. $n = 2$ biological replicates, each of which had 3 technical replicates. **g,** The effect of retinoic acid (RA) and testosterone (TS) on the expression of a late PGC maker (*Mvh*), early meiotic markers (*Stra8* and *Sycp3*), and post-meiotic markers (*Haprin* and *Acrosin*). 3 biological replicates, each of which had 3 technical replicates, were used for two-sided *t*-test. $^{**}P < 0.01$, $^{*}P < 0.05$. DAPI (blue) for nuclear staining. Scale bar, 50 μm.

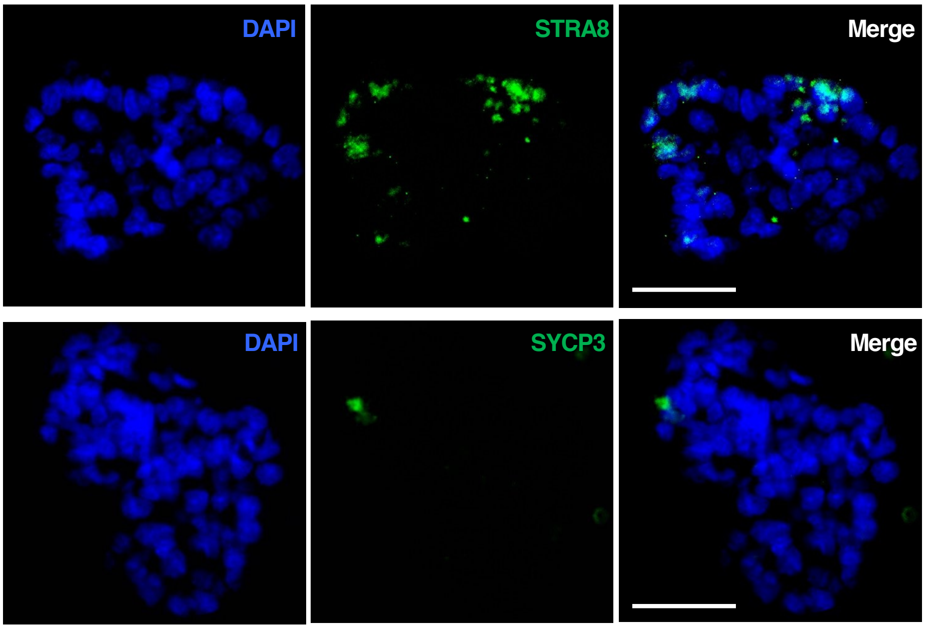

Supplementary Fig. 5. Detection of early meiotic cells. Immunocytochemistry for early meiotic markers, STRA8 (top, green) and SYCP3 (bottom, green) in day 12 secondary testicular organoids. DAPI (blue) for nuclear staining. Scale bar, 50 μm.

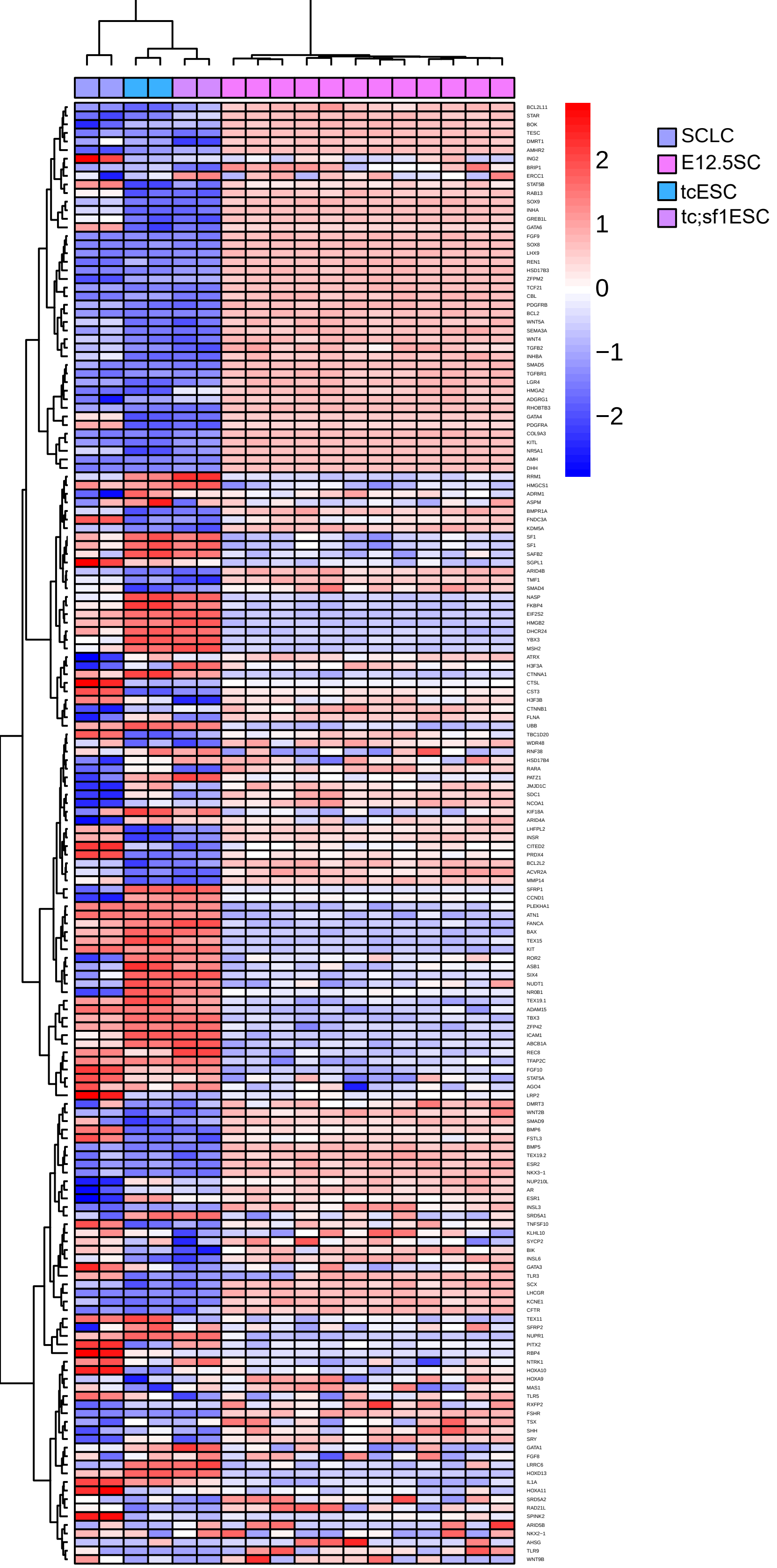
SCLC
E12.5SC
tcESC
tc;sf1ESC
2
1
0
−1
−2
BCL2L11
STAR
BOK
TESC
DMRT1
AMHR2
ING2
BRIP1
ERCC1
STAT5B
RAB13
SOX9
INHA
GREB1L
GATA6
FGF9
SOX8
LHX9
REN1
HSD17B3
ZFPM2
TCF21
CBL
PDGFRB
BCL2
WNT5A
SEMA3A
WNT4
TGFB2
INHBA
SMAD5
TGFBR1
LGR4
HMGA2
ADGRG1
RHOBTB3
GATA4
PDGFRA
COL9A3
KITL
NR5A1
AMH
DHH
RRM1
HMGCS1
ADRM1
ASPM
BMPR1A
FNDC3A
KDM5A
SF1
SF1
SAFB2
SGPL1
ARID4B
TMF1
SMAD4
NASP
FKBP4
EIF2S2
HMGB2
DHCR24
YBX3
MSH2
ATRX
H3F3A
CTNNA1
CTSL
CST3
H3F3B
CTNNB1
FLNA
UBB
TBC1D20
WDR48
RNF38
HSD17B4
RARA
PATZ1
JMJD1C
SDC1
NCOA1
KIF18A
ARID4A
LHFPL2
INSR
CITED2
PRDX4
BCL2L2
ACVR2A
MMP14
SFRP1
CCND1
PLEKHA1
ATN1
FANCA
BAX
TEX15
KIT
ROR2
ASB1
SIX4
NUDT1
NR0B1
TEX19.1
ADAM15
TBX3
ZFP42
ICAM1
ABCB1A
REC8
TFAP2C
FGF10
STAT5A
AGO4
LRP2
DMRT3
WNT2B
SMAD9
BMP6
FSTL3
BMP5
TEX19.2
ESR2
NKX3−1
NUP210L
AR
ESR1
INSL3
SRD5A1
TNFSF10
KLHL10
SYCP2
BIK
INSL6
GATA3
TLR3
SCX
LHCGR
KCNE1
CFTR
TEX11
SFRP2
NUPR1
PITX2
RBP4
NTRK1
HOXA10
HOXA9
MAS1
TLR5
RXFP2
FSHR
TSX
SHH
SRY
GATA1
FGF8
LRRC6
HOXD13
IL1A
HOXA11
SRD5A2
RAD21L
SPINK2
ARID5B
NKX2−1
AHSG
TLR9
WNT9B

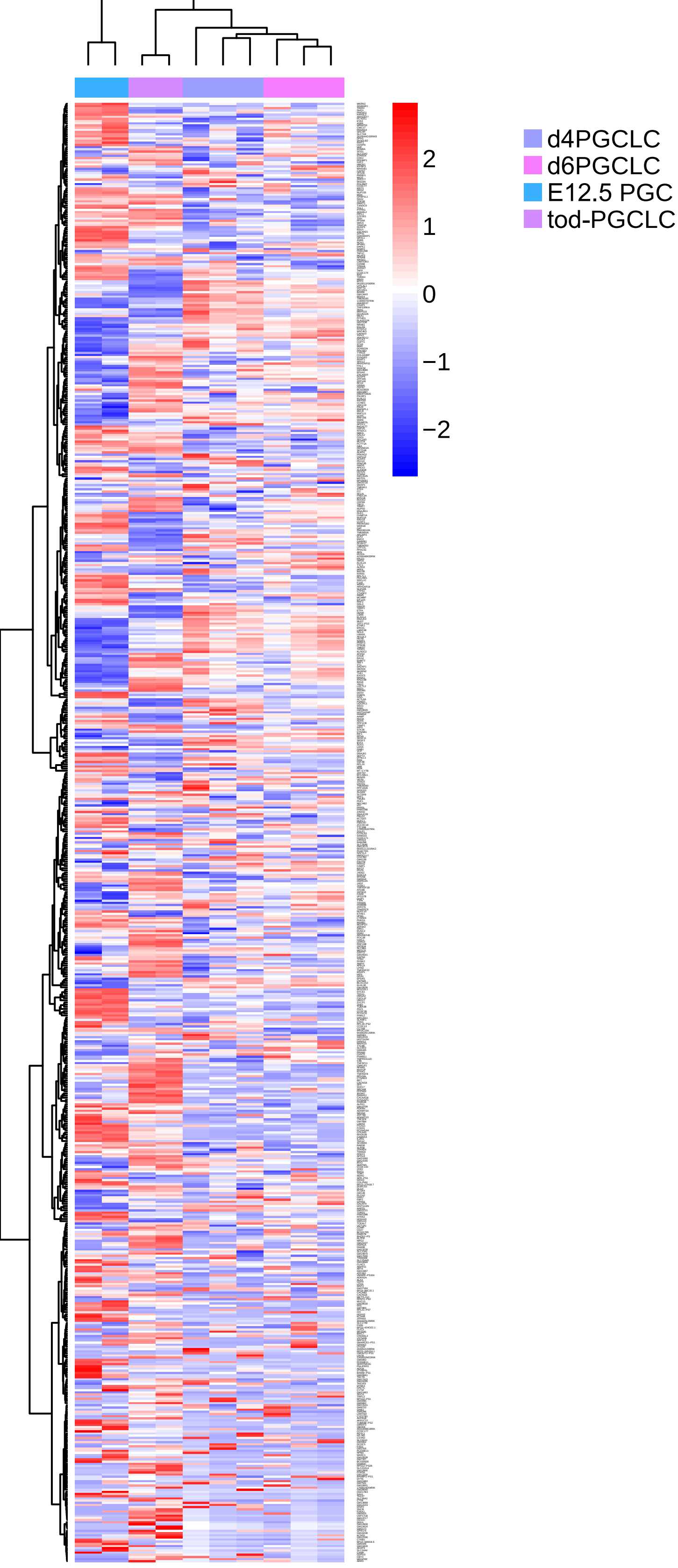
2
1
0
−1
−2
d4PGCLC
d6PGCLC
E12.5 PGC
tod-PGCLC

Supplementary Fig. 6. Comparison of selected transcriptomes of SCLCs and tod-PGCLCs. a.b, Heatmap of the expression levels of GO-selected DEGs which were associated with either 'sex determination', 'sex differentiation' 'Sertoli cell development' or 'Sertoli cell differentiation' for tcESCs, tc;Sf1ESCs, SCLCs and E12.5SCs (**a**), and either 'germ cell development' or 'male gamete generation' for d4PGCLCs, d6PGCLCs, tod-PGCLCs and E12.5PGCs (**b**).

a

	Gene name	Mean base read	tc;sf1ESC vs tcESC	
			log2 FC	*P* adjust
Pluripotent stem cell	Nr5a1	3285.31	6.41157	4.35E-05
	Dkk1	146.62	2.21555	2.35E-10
	Gata6	782.35	1.41802	5.56E-05
	Cdx2	20.09	1.32690	4.03E-02
	Sall1	603.6	1.18897	1.51E-64
	Lin28a	611.59	-1.24605	1.92E-13
	Fgf2	87.89	-1.26573	9.84E-05
	Foxp1	222.81	-1.82498	2.18E-34
	Eomes	67.59	-1.84064	1.13E-15

b

	Gene name	Mean base read	SCLC vs tcESC		SCLC vs tc;sf1ESC		SCLC vs E12.5SC	
			log2 FC	*P* adjust	log2 FC	*P* adjust	log2 FC	*P* adjust
Sertoli cell	Wt1	3170.91	1.21090	1.29E-04	1.78282	1.06E-06	-8.00780	0.00E+00
	Sox9	1182.05	5.01401	3.63E-21	5.46017	4.15E-19	-4.54544	1.09E-116
	Nr5a1	3285.31	9.68729	2.68E-11	3.27572	5.24E-71	-4.65752	0.00E+00
	Gata4	4551.89	7.55158	0.00E+00	5.91694	0.00E+00	nd	-
	Dmrt1	600.42	0.72403	5.92E-03	2.24756	2.21E-18	-1.42464	1.74E-14
	Sox8	521.75	-0.27664	*5.62E-01	0.86575	*8.26E-02	-6.68845	1.46E-152
	Sox10	336.77	-0.81707	*3.97E-01	0.12893	*9.72E-01	-8.56268	4.81E-46
	Amh	13375.36	-0.12330	*8.40E-01	-0.49227	*2.44E-01	-11.46285	0.00E+00
	Dhh	3603.66	-1.07786	*2.18E-01	-2.67801	7.77E-05	-11.98446	1.56E-90
	Fgf9	455.07	0.50787	*6.46E-01	4.47754	7.20E-03	-8.41415	1.77E-66
	Gdnf	663.63	4.31055	6.38E-03	5.69981	3.00E-04	-7.73561	1.67E-127
	Gfra1	186.27	6.41247	4.10E-13	7.93979	1.37E-07	-2.16774	1.65E-33
	Vnn1	1924.50	4.82784	3.00E-138	6.26365	1.86E-112	-2.12375	1.59E-108
	Ptgds	2584.06	6.56556	7.30E-06	5.07292	1.16E-03	-8.88251	6.05E-288
	Cyp26b1	3680.17	2.50616	1.96E-17	3.45084	1.76E-18	-7.01274	0.00E+00
	Hsd17b3	893.49	-2.51950	*2.43E-01	-4.38274	9.76E-03	-13.99983	1.28E-21
	Cited2	1227.50	2.53445	7.85E-57	3.40166	8.21E-99	1.82979	1.12E-51
	Insr	987.82	2.65939	2.20E-172	1.90630	8.77E-96	nd	-
Granulosa cell	Wnt4	160.18	3.81595	8.06E-15	4.63257	4.24E-13	-2.68719	1.53E-53
	Nr0b1	452.12	-5.44125	7.30E-279	-4.73951	6.99E-210	-2.62664	6.27E-78
	Esr1	73.62	0.55227	*4.63E-01	0.95484	*2.08E-01	-4.85098	3.40E-39
	Esr2	30.69	-1.30455	*3.06E-01	-0.13095	*9.75E-01	-6.09129	3.48E-12
	Fst	282.52	1.32522	8.23E-03	1.74721	3.48E-04	2.73970	1.89E-14
	Rspo1	100.20	-1.34926	1.54E-06	0.52374	*1.96E-01	-3.48532	1.03E-53

c

	Gene name	Mean base read	todPGCLC vs PGCLCd4		todPGCLC vs PGCLCd6		todPGCLC vs E12.5PGC	
			log2FC	*P* adjust	log2FC	*P* adjust	log2FC	*P* adjust
ES	Sox2	2557.34	1.16417	1.68E-04	1.63447	1.29E-07	4.40433	1.28E-42
	Nanog	2254.22	0.84486	*9.31E-02	1.83736	1.37E-04	2.35253	3.21E-06
	Klf4	146.67	2.41287	3.46E-05	2.65994	7.07E-06	3.40053	4.33E-08
ICM	Prdm14	410.66	-0.11305	*8.02E-01	-0.42329	*3.21E-01	-0.46483	*2.80E-01
	Zfp42	2120.42	-0.68411	*2.12E-01	-0.07024	*9.25E-01	0.16933	*8.02E-01
	Esrrb	1221.30	2.23504	1.06E-09	2.01774	8.11E-08	3.80502	4.35E-22
	Klf2	2205.68	-1.12725	1.69E-02	-0.66834	*2.02E-01	5.44128	1.47E-28
Epiblast	Wnt3	23.90	-2.72770	1.45E-03	-3.94935	2.38E-19	-5.47874	3.88E-34
	Fgf5	45.64	2.13557	5.14E-04	0.41715	*5.48E-01	1.47566	1.71E-02
	Dnmt3b	428.82	2.20995	2.67E-03	2.28235	2.51E-03	4.33178	2.16E-08
early PGC	Utf1	3286.32	-0.68734	3.08E-02	-1.30761	2.26E-05	-0.52870	*1.37E-01
	Prdm1	823.40	1.35299	3.46E-04	0.45218	*3.06E-01	1.22044	3.19E-03
	Tfap2c	2250.70	-1.04296	2.07E-05	-1.22779	7.16E-07	2.46705	3.86E-21
	Nanos3	195.83	-3.01579	9.05E-08	-3.67815	7.60E-11	-3.53859	5.01E-09
	Kit	3009.05	0.01858	*9.65E-01	-1.08250	8.76E-04	-1.31386	1.16E-04
	Dnd1	894.94	-1.70844	1.92E-07	-1.02048	3.79E-03	-1.99152	2.08E-08
late PGC	Tdrd5	374.70	-1.66235	3.15E-03	-1.49440	1.09E-02	-0.22448	*7.61E-01
	Dnmt1	2597.37	1.05888	2.89E-05	0.47397	*9.33E-02	-0.73081	9.73E-03
	Dnmt3a	653.62	2.33581	1.18E-14	1.43338	3.97E-06	-0.25918	*4.94E-01
	Dazl	2736.78	0.29383	*5.66E-01	1.85248	3.51E-05	-3.35379	2.41E-13
Meiosis	Sycp3	123.66	-1.14776	3.51E-02	0.49786	*4.52E-01	-2.78240	2.70E-07
	Stra8	135.22	4.10551	4.19E-02	3.26225	*1.30E-01	7.08861	9.15E-04
	Mnd1	612.34	-2.45586	7.76E-24	-0.78624	4.07E-03	-0.94636	7.59E-04
	Syce1	183.84	-0.89267	2.18E-02	0.89078	4.00E-02	-2.80553	1.14E-13
	Dmc1	97.17	0.67122	*1.40E-01	1.32337	2.88E-03	1.12979	1.40E-02
	Sycp1	84.38	1.54394	3.08E-03	1.98277	1.67E-04	-2.79275	5.03E-11
	Rec8	222.15	1.64687	2.07E-05	1.40390	4.13E-04	3.74134	2.13E-19

Supplementary Table 1. Selected DEGs that may be essential for SCLC and PGCLC differentiation. a-c, The fold expression comparison of genes essential for pluripotent stem cells (**a**), Sertoli cells and ovarian granulosa cells (**b**), and the process of germ cell development (**c**), which were selected from Extended Data Figure 6. Asterisks indicate that values are not statistically significant.

Medium base

MEIM	DMEM/F12+Glutamax, 1x B27 (-vitamin A), 1x penicillin-streptomycin
RAG	DMEM/F12+Glutamax, 1x B27 (-vitamin A), 1x penicillin-streptomycin<br>1x non-essential amino acids, 0.1 mM β-mercaptoethanol

3-step TesLC induction

	Medium	Base	Supplements
Step 1	MEIM	MEIM	3 μM CHIR99021, 1 μM TTNPB, 10 μM Y27632
Step 2	MEIM	MEIM	3 μM CHIR99021
Step 3	RAG	RAG	3 μM CHIR99021, 10 μM Y27632, 100 ng/ml BMP7

2-step TesLC induction

	Medium	Base	Supplements
Step 1	MEIM	MEIM	3 μM CHIR99021, 1 μM TTNPB, 10 μM Y27632*
Step 2	RAG+C	RAG	3 μM CHIR99021
	RAG+CBF	RAG	3 μM CHIR99021, 1 μM TTNPB, 100 ng/ml BMP7
	RAG+CBF+IG	RAG	3 μM CHIR99021, 1 μM TTNPB, 100 ng/ml BMP7<br>100 ng/ml IGF1, 20 ng/ml GDNF
	RAG+CBF+D	RAG	3 μM CHIR99021, 1 μM TTNPB, 100 ng/ml BMP7<br>100 ng/ml DHH

* optional

Testicular organoid induction

	Medium	Base	Supplements
Primary / Secondary	RAG+C	RAG	3 μM CHIR99021
	RAG+C+SSC	RAG	3 μM CHIR99021, 12 ng/ml bFGF, 20 ng/ml GDNF<br>50 ng/ml EGF, 1000 U/ml LIF
	RAG+C+RA	RAG	3 μM CHIR99021, 1 μM retinoic acid
	RAG+C+TS	RAG	3 μM CHIR99021, 10 μM testosterone

Supplementary Table 2. Media components.

Gene	Forward Primer	Reverse Primer	Reference
Sox2	CATGAGAGCAAGTACTGGCAAG	CCAACGATATCAACCTGCATGG	Kurimoto *et al*. (2006)[1]
Nanog	CTTTCACCTATTAAGGTGCTTGC	TGGCATCGGTTCATCATGGTAC	
Oct4	GATGCTGTGAGCCAAGGCAAG	GGCTCCTGATCAACAGCATCAC	
Gapdh	ATGAATACGGCTACAGCAACAGG	CTCTTGCTCAGTGTCCTTGCTG	
Osr1	GACCGCGGCGGAACAAGATA	CACTGTGGGCAGGCCATTCA	Oeda *et al*. (2013)[2]
Lhx1	TGGACCGTTTCCTCTTGAAC	TGTTCTCTTTGGCGACACTG	Nishikawa *et al*. (2012)[3]
Pax2	AGGAAACGCGAGGAAGATGT	ACATCGGGATAGGAAGGACG	Jeon *et al*. (2012)[4]
Sox9	AAGAAAGACCACCCCGATTACA	CAGCGCCTTGAAGATAGCATT	Bouma *et al*. (2004)[5]
Gata4	CCTGGAAGACACCCCAATCTC	AGGTAGTGTCCCGTCCCATCT	
Wt1	CGGTCCGACCATCTGAAGAC	GTTGTGATGGCGGACCAATT	
Sf1	CGCAACAACCTTCTCATTGAGA	TGGATCCCTAATGCAAGGAGTCT	
Dmrt1	GTGCCTGCTCAGACTGGAAAC	GATCTGGGACATGCTCTGGC	
Vnn1	TGGCCAAGAACAACTCCATCT	CACCACATCAGTGTTGTACTGGAAT	
Amh	CTATTTGGTGCTAACCGTGGACTT	AAGGCTTGCAGCTGATCGAT	
Dhh	ACCCCGACATAATCTTCAAGGAT	GTACTCCGGGCCACATGTTC	
Star	TCTCTAGTGTCTCCCACTGCATAGC	TTAGCATCCCCTGTTCGTAGCT	
Fshr	GGCCAGGTCAACATACCGCTTG	TGCCTTGAAATAGACTTGTTGCAAATTG	O’Shaughnessy *et al*. (2008)[6]
Hsd3b6	GCTCCAGACTGGGACTGCTGACAC	AATCCTCTGGCCCAAAAACCCTC	
Hsd3b1	CAAGTGTGCCAGCCTTCATCT	TTCATGATTCTGTTCCTCGTGG	Inoue *et al*. (2016)[7]
Hsd17b3	ATGGAGTCAAGGAGGAAAGGC	GGCTGTAAAGAGGCCAGGG	
Couptf-II	TGCGGAGGAACCTGAGCTAC	CTGTACAGCTTCCCGTCTCAT	
Nrf2	CAGTGCTCCTATGCGTGAA	GCGGCTTGAATGTTTGTC	
Cyp11a1	TGGCCCCATTTACAGGGAGAA	GGCATCTGAACTCTTAAACAGGA	
Foxd1	AGTCTCCAAGAAATGCCTCTACT	TTCCGCATGTCCATTATGATACAA	
Hoxd11	CCGCAGCCTCTAACTTCTACA	GCCTCGTAGAACTGATCAAAGC	Montavon *et al*., (2008)[8]
Six2	AGGAAAGGGAGAACAGCGA	CGTCTTCTCATCCTCGGAAC	
Cited2	AGACGGAAGGACTGGAAATG	GCTGCTGCTGGTGATGAT	
Wnt3	CAAGCACAACAATGAAGCAGGC	TCGGGACTCACGGTGTTTCTC	Hayashi *et al*. (2011)
Fgf5	AAAGTCAATGGCTCCCACGAA	CTTCAGTCTGTACTTCACT	
Dnmt3b	GACTCGCGTGCAATAACCTTAG	GGTCACTTTCCCTCACTCTGG	
Prdm1	AGCATGACCTGACATTGACACC	CTCAACACTCTCATGTAAGAGGC	
Prdm14	ACAGCCAAGCAATTTGCACTAC	TTACCTGGCATTTTCATTGCTC	
Tcfap2c	GGGCTTTTCTCTCTTGGCTGGT	TCCACACGTCACCCACACAA	
Dnd1	CCCTAAATGGGTTAAGCAGAGC	GGCAAGGTTCCTCACAACTAAAG	
Vasa	TATGTGCCTCCCAGCTTCAGTA	CTGGATTGGGAGCTTGTGAAGA	
Dazl	TCCTTGACTTGTGGTTGCTG	CCACCTTCGAGGTTTTACCA	
Nanos3	CACTACGGCCTAGGAGCTTGG	TGATCGCTGACAAGACTGTGG	
Plzf	CTTCACTTGCCTCCAGTCCAGA	TACACAGAAGGAAGGCAGGTGT	Ishikura *et al*. (2016)
Id4	TCCCTTGCAGAGCTTTTGCTAT	ACCAGAGAGCTGTTACCTCTGA	
cKit	CAGTTACCGCGCTCTGTTTG	GCCCCTTAAGTACCTGACATCC	
Gfra1	TTTTACTGACAGTTGCGTCCAC	TGAATGTGCTTCTGCTCAAAGTG	
Stra8	GTTTCCTGCGTGTTCCACAAG	CACCCGAGGCTCAAGCTTC	
Sycp3	AGCAGAGAGCTTGGTCGGG	TCCGGTGAGCTGTCGCTGTC	Dong *et al*. (2017)[9]
Acrosin	CGGAGTCTACACAGCCACCT	GCATGAGTGATGAGGAGGTT	Geijsen *et al*. (2004)[10]
Haprin	CCAGAACATGAGACAGAGAG	AGCAACTTCCTGAGCATACC	
Sox17	CCGATGAACGCCTTTATGGTG	GGTCAACGCCTTCCAAGACTT	Mfopou *et al*. (2007)[11]

Supplementary Table 3. Primers used for quantitative RT-PCR.

Supplementary References

1. Kurimoto, K. *et al*. An improved single-cell cDNA amplification method for efficient high-density oligonucleotide microarray analysis. *Nucleic Acids Res.* **34**, e42 (2006).

2. Oeda, S. *et al*. Induction of intermediate mesoderm by retinoic acid receptor signaling from differentiating mouse embryonic stem cells. *Int. J. Dev. Biol.* **57**, 383-389 (2013).

3. Nishikawa, M. *et al*. Stepwise renal lineage differentiation of mouse embryonic stem cells tracing in vivo development. *Biochem. Bioph. Res. Co.* **417**, 897-902 (2012).

4. Jeon, K. *et al*. Bax inhibitor-1 enhances survival and neuronal differentiation of embryonic stem cells via differential regulation of mitogen-activated protein kinases activities. *Biochim. Biophys. Acta* **1823**, 2190-2200 (2012).

5. Bouma, G. J. et al. Using real time RT-PCR analysis to determine multiple gene expression pattern during XX and XY mouse fetal gonadal development. *Gene Expr. Patterns* **9**, 141-149 (2004).

6. O'Shaughnessy, P. J., Hu, L. & Baker, P. J. Effect of germ cell depletion on levels of specific mRNA transcripts in mouse Sertoli cells and Leydig cells. *Reproduction* **135**, 839-850 (2008).

7. Inoue, M. *et al*. Isolation and characterization of fetal Leydig progenitor cells of male mice. *Endocrinology*, **157**, 1222-1233 (2016).

8. Montavon, T., Le Garrec, J. F., Kerszberg, M. & Duboule, D. Modeling Hox gene regulation in digits: reverse collinearity and the molecular origin of thumbness. *Genes Dev.* **22**, 346-359 (2008).

9. Dong, G. *et al*. Retinoic acid combined with spermatogonial stem cell conditions facilitate the generation of mouse germ-like cells. *Biosci. Rep.* **37**, BSR20170637 (2017).

10. Geijsen, N. et al. , Derivation of embryonic germ cells and male gametes from embryonic stem cells. *Nature* **427**, 148-154 (2004).

11. Mfopou, J. K. *et al*. Sonic hedgehog and other soluble factors from differentiating embryoid bodies inhibit pancreas development. *Stem Cells* **25**,1156-1165 (2007).
