# Supplementary material for: Testicular somatic cell-like cells derived from embryonic stem cells induce differentiation of epiblasts into germ cells
Source: Commun Biol. 2021 Jun 28;4:802. doi: 10.1038/s42003-021-02322-8 (PMC8239049; doi:10.1038/s42003-021-02322-8)
Supplement: Supplementary file 3 — Description of Additional Supplementary Files [file 42003_2021_2322_MOESM3_ESM.pdf]

Description of Additional Supplementary Files

File name: Supplementary Data 1

Description: Lists of differentially expressed genes (DEGs). **a-f,** TPM normalised reads were compared between tcESCs and tc;sf1ESCs (**a**), tcESCs and SCLCs (**b**), tcESCs and E12.5SCs (**c**), tc;sf1ESCs and SCLCs (**d**), tc;sf1ESCs and E12.5SCs (**e**), and SCLCs and E12.5SCs (**f**). DEGs were defined by log fold change (LFC) >1 or <-1 and adjusted p-value (Padj) ≤0.05. The lists are ranked by LFC.

File name: Supplementary Data 2

Description: The results of GO enrichment analysis. **a-f,** Top 10 GO terms enriched in comparison between tcESCs and tc;sf1ESCs (**a**), tcESCs and SCLCs (**b**), tcESCs and E12.5SCs (**c**), tc;sf1ESCs and SCLCs (**d**), tc;sf1ESCs and E12.5SCs (**e**), and SCLCs and E12.5SCs (**f**).

File name: Supplementary Data 3

Description: Data source underlying graphs in the main figures.
