# Supplementary material for: Testicular somatic cell-like cells derived from embryonic stem cells induce differentiation of epiblasts into germ cells
Source: Commun Biol. 2021 Jun 28;4:802. doi: 10.1038/s42003-021-02322-8 (PMC8239049; doi:10.1038/s42003-021-02322-8)
Supplement: Supplementary file 7 — Reporting Summary [file 42003_2021_2322_MOESM7_ESM.pdf]

Corresponding author(s): Dr. Ryohei Sekido

Last updated by author(s): Jun 9, 2021

Reporting Summary

Nature Research wishes to improve the reproducibility of the work that we publish. This form provides structure for consistency and transparency in reporting. For further information on Nature Research policies, see our Editorial Policies and the Editorial Policy Checklist.

Statistics

For all statistical analyses, confirm that the following items are present in the figure legend, table legend, main text, or Methods section.

n/a	Confirmed	
☐	☒	The exact sample size (*n*) for each experimental group/condition, given as a discrete number and unit of measurement
☐	☒	A statement on whether measurements were taken from distinct samples or whether the same sample was measured repeatedly
☐	☒	The statistical test(s) used AND whether they are one- or two-sided<br>*Only common tests should be described solely by name; describe more complex techniques in the Methods section.*
☒	☐	A description of all covariates tested
☐	☒	A description of any assumptions or corrections, such as tests of normality and adjustment for multiple comparisons
☐	☒	A full description of the statistical parameters including central tendency (e.g. means) or other basic estimates (e.g. regression coefficient) AND variation (e.g. standard deviation) or associated estimates of uncertainty (e.g. confidence intervals)
☐	☒	For null hypothesis testing, the test statistic (e.g. *F*, *t*, *r*) with confidence intervals, effect sizes, degrees of freedom and *P* value noted<br>*Give P values as exact values whenever suitable.*
☒	☐	For Bayesian analysis, information on the choice of priors and Markov chain Monte Carlo settings
☒	☐	For hierarchical and complex designs, identification of the appropriate level for tests and full reporting of outcomes
☒	☐	Estimates of effect sizes (e.g. Cohen's *d*, Pearson's *r*), indicating how they were calculated

Our web collection on statistics for biologists contains articles on many of the points above.

Software and code

Policy information about availability of computer code

Data collection	NCBI (Genome assembly GRCm38/mm10, and the sequence read archive (SRA)), Eurofins Genomics (High-throughput RNA sequencing), BD LSR II (Flow cytometry), and Zeiss Axio Observer Z1 (fluorescence microscopy)
Data analysis	DESeq2 (Transcriptome analysis), WebGestalt (GO enrichment), GSEA and EMBL-EBI QuickGO (GO search), StepOnePlus (real-time PCR), Image J and Zen (digital imaging), PHERAstar FSX (ELISA) and Flowjo (Flow cytometry)

For manuscripts utilizing custom algorithms or software that are central to the research but not yet described in published literature, software must be made available to editors and reviewers. We strongly encourage code deposition in a community repository (e.g. GitHub). See the Nature Research guidelines for submitting code & software for further information.

Data

Policy information about availability of data

All manuscripts must include a data availability statement. This statement should provide the following information, where applicable:

- Accession codes, unique identifiers, or web links for publicly available datasets
- A list of figures that have associated raw data
- A description of any restrictions on data availability

RNA-seq data that support the findings of this study have been deposited in the NCBI Gene Expression Omnibus (GEO) with the GEO accession codes “GSE149932”.

Field-specific reporting

Please select the one below that is the best fit for your research. If you are not sure, read the appropriate sections before making your selection.

☒ Life sciences ☐ Behavioural & social sciences ☐ Ecological, evolutionary & environmental sciences

For a reference copy of the document with all sections, see nature.com/documents/nr-reporting-summary-flat.pdf

Life sciences study design

All studies must disclose on these points even when the disclosure is negative.

Sample size	Sample size was estimated from similar studies that were published in scientific journals previously. These studies have been cited in the manuscript, notably reference #1, 2, 3, 9, 10, 11, 12, 13.
Data exclusions	We excluded data showing no statistically significance, notably p-value and adjusted p-value below 0.05.
Replication	We biologically replicated all our experiments. We also technically replicated when we ran samples on devices or instruments.
Randomization	This work was designed as a PhD project supported by BBSRC EASTBIO doctoral training partnership. The main contributor, i.e. the first author, was selected from the public when I advertised the project. I did not choose a particular batch of ES cell lines that are the main materials in this project, because they were gifted from MRC National Institute for Medical Research and the University of Cambridge. They would have been arbitrarily selected by the senders.
Blinding	I planed and designed this work designed as an in-house project at the University of Aberdeen. Because all the participants in Aberdeen worked as colleagues on a daily basis, blinding was not relevant to this study. However, high-throughput transcriptome analysis was performed by Dr Owen at the University College London who has been blinded to the Aberdeen participants except me.

Reporting for specific materials, systems and methods

We require information from authors about some types of materials, experimental systems and methods used in many studies. Here, indicate whether each material, system or method listed is relevant to your study. If you are not sure if a list item applies to your research, read the appropriate section before selecting a response.

Materials & experimental systems

n/a	Involved in the study	
☐	☒	Antibodies
☐	☒	Eukaryotic cell lines
☒	☐	Palaeontology and archaeology
☒	☐	Animals and other organisms
☒	☐	Human research participants
☒	☐	Clinical data
☒	☐	Dual use research of concern

Methods

n/a	Involved in the study	
☒	☐	ChIP-seq
☐	☒	Flow cytometry
☒	☐	MRI-based neuroimaging

Antibodies

Antibodies used	anti-active caspase 3 (Abcam, ab2302), anti-FGF5 (Proteintech, 18171-1-AP), anti-GATA4 (SantaCruz, sc-1237), anti-GFP (Abcam, ab13970), anti-HSD3B (gift from Ian. Mason), anti-Ki67 (BioLegend, 652401), anti-laminin (gift from Harold Erickson), anti-MVH Novus Biologicals, NBP2-24558), anti-OSR1 (Abcam, ab230627), anti-Pax2 (Novus Biologicals NPB2-57700), anti-SOX2 (Novus Biologicals, AF2018), anti-SOX9 (Novus Biologicals, AF3075), anti-SF1/NR5A1 (Novus Biologicals NPB1-52823), anti-StAR (Biorbyt, orb7014), anti-STRA8 (Abcam, ab49602), and anti-SYCP3 (Novus Biologicals, NB300-230).
Validation	All the antibodies have been validated in manufactures' website or published papers.

Eukaryotic cell lines

Policy information about cell lines

Cell line source(s)	Mouse embryonic stem cell line tcESC was generated from a transgenic mouse strain on C57BL6 background at MRC National Institute for Medical Research, UK. Mouse embryonic stem cell line Prdm1-gfp ESC was generated in Azim Surani's laboratory at the University of Cambridge.
Authentication	Both cell lines have been authenticated by MRC and the University of Cambridge.

Mycoplasma contamination	Both cell lines have been tested negative for mycoplasma contamination.
Commonly misidentified lines (See ICLAC register)	We did not used any misidentified cell lines.

Flow Cytometry

Plots

Confirm that:

- [x] The axis labels state the marker and fluorochrome used (e.g. CD4-FITC).
- [x] The axis scales are clearly visible. Include numbers along axes only for bottom left plot of group (a 'group' is an analysis of identical markers).
- [x] All plots are contour plots with outliers or pseudocolor plots.
- [x] A numerical value for number of cells or percentage (with statistics) is provided.

Methodology

Sample preparation	Cultured cells and/or organoids were dissociated with a mixture of 1x Accutase, 0.1% collagenase IV and 0.1% dispase, and filtered them through a 40 micron cell strainer.
Instrument	BD LSR II
Software	Flowjo
Cell population abundance	The purity of SCLCs was identified as the cells positive for CFP-fluorescence that is derived from a cfp transgene integrated in the SCLC genome. The purity of PGCLCs was identified as the cells double-positive forCD49f-PE and GFP-fluorescence that is derived from a gfp transgene integrated in the PGCLC genome. The abundance of these cells was determined by preliminary flow cytometry analyses. The analysis of 500,000 events provided 3.7% of purity for SCLCs and 2% for PGCLCs. For sorting, several millions of these cells were prepared and a few thousands were isolated.
Gating strategy	After preliminary gating with SSC/FSC, singlet cells were selected by FSC / Trigger plus width. CFP-positive cells were gated through SSC / A460/50(405). Then, to remove propidium iodide-positive dead cells, the CFP-positive cell population was gated through A 692/40(488) / A460/50(405). For GFP-positive PGCLCs, singlet cells were selected as described above. Dead cells labelled with DAPI were removed through SSC / DAPI gating from singlet cell population. PGCLCs labelled as GFP fluorescence and CD49f-PE double positive cells were gated through PE-A / FITC-A.

- [x] Tick this box to confirm that a figure exemplifying the gating strategy is provided in the Supplementary Information.
